# Prediction and verification of novel peptide targets of protein tyrosine phosphatase 1B

**DOI:** 10.1016/j.bmc.2016.03.030

**Published:** 2016-08-01

**Authors:** Xun Li, Maja Köhn

**Affiliations:** a European Molecular Biology Laboratory, Genome Biology Unit, Meyerhofstrasse 1, 69117 Heidelberg, Germany; b European Molecular Biology Laboratory-European Bioinformatics Institute, Wellcome Trust Genome Campus, Hinxton, Cambridge CB10 1SD, United Kingdom

**Keywords:** Protein tyrosine phosphatases, Computational prediction, Peptide substrates, PTP1B

## Abstract

Phosphotyrosine peptides are useful starting points for inhibitor design and for the search for protein tyrosine phosphatase (PTP) phosphoprotein substrates. To identify novel phosphopeptide substrates of PTP1B, we developed a computational prediction protocol based on a virtual library of protein sequences with known phosphotyrosine sites. To these we applied sequence-based methods, biologically meaningful filters and molecular docking. Five peptides were selected for biochemical testing of their potential as PTP1B substrates. All five peptides were equally good substrates for PTP1B compared to a known peptide substrate whereas appropriate control peptides were not recognized, showing that our protocol can be used to identify novel peptide substrates of PTP1B.

## Graphical abstract

**Figure:**
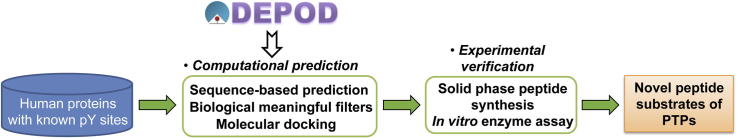

## 1 Introduction

To date, the knowledge of phosphatase substrates is far less than that of kinase substrates, and the identification of substrates is one of the key challenges in phosphatase research.^1^ Peptides can play a crucial role in finding new substrates.^2^ Furthermore, they can be used as starting point for chemical tool development, for example for inhibitors or pull-down baits.^3^ New peptide substrates can be identified, for example, by using phosphopeptide microarrays^2^, ^4^ or peptide libraries.^5^ Computational methods are a cheap alternative to the aforementioned approaches. They have so far been applied to find protein substrates for phosphatases,^6^ and for analysis in combination with peptide microarrays.^2^ Here, we have developed a computational protocol for identifying human protein derived peptide substrates of protein-tyrosine phosphatase 1B (PTP1B) as a model phosphatase. PTP1B is a well-studied phosphatase involved in cancer and diabetes.^7^ Our method led to the discovery of five biochemically confirmed novel peptide substrates of PTP1B.

## 2 Results and discussion

As starting point we constructed a virtual library composed of human proteins with known phosphotyrosine (pY) sites but not containing artificial pY-containing sequences. Phospho.ELM,^8^ PhosphoSitePlus^9^ and HPRD^10^ were used for this purpose. There are in total 3799 non-redundant protein sequences in the library, and 4931 non-redundant 11-mer peptides (with pY in the middle) were extracted. The length of 11 amino acids was chosen following previous studies testing PTP phosphopeptide substrate specificity.(b), 11

**Figure 1:**
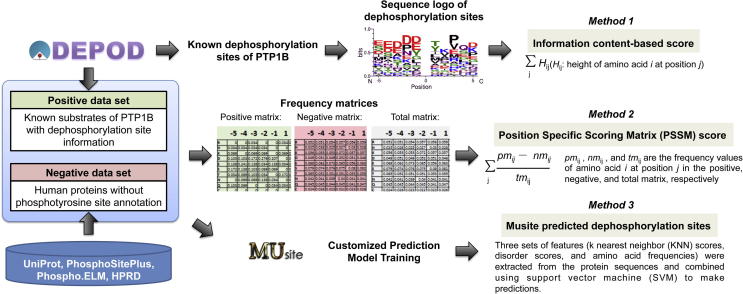
Schematic flow-chart of the sequence-based prediction of dephosphorylation sites of PTP1B.

A sequence-based prediction that employs three methods (Fig. 1) was then carried out on these sequences. These methods are based on the dephosphorylation data from the human DEPhOsphorylation Database DEPOD.^1^, ^12^ While method 2 has been used before for PTP1B substrate identification,2b the other two methods have not, and they employ different algorithms than method 2. We reasoned that the consensus prediction result from all three methods would give us the best result for finding PTP1B peptide substrates.

The 4931 extracted pY-containing peptides were assigned information content-based scores (method 1) and also position specific scoring matrix (PSSM) scores2b (method 2) and ranked accordingly. The 3799 protein sequences, which contain 89874 tyrosines in total, were given to the pre-trained prediction model customized for PTP1B in Musite.^13^ 5825 tyrosines of the 89874 were predicted to be dephosphorylation sites for PTP1B (specificity ⩾ 95%) (method 3). The top 10% peptides were taken from each method. The three predictions were combined together to get a common set, which are 139 non-redundant pY-containing peptides. They correspond to 231 pY sites on 191 original protein sequences (122 genes). Among them 15 genes code known protein substrates of PTP1B, and for 12 of them our predicted dephosphorylation sites match with reported ones (Supplementary Table S1). For the remaining 3 protein substrates dephosphorylation sites are unknown. DEPOD contains 39 substrates, and we did not identify 24 of those. Reasons for this lie in the strict cut-off that we applied to obtain the consensus results from the three methods of the sequence-based prediction. For example, the PTP1B substrate Src^14^ was predicted as good substrate only by method 3 (Musite cut-off 95%, sequence score for STEPQpYQPGEN 98.48%), but not by methods 1 (rank 803/4931) and 2 (rank 985/4931). Since a strict cut-off generally decreases the chances of finding false positives and since we did identify dephosphorylation sites of 13 proteins as well as 107 novel substrate candidates, we judged the result of our cut-off setting as acceptable.

Next, we applied biologically meaningful filters which are derived from DEPOD to the predicted substrates. The candidate proteins should be either the known substrates of certain PTPs that share common known substrates with PTP1B, or they should be mapped within the same KEGG^15^ and NCI-Nature PID^16^ pathways as PTP1B. The filtering resulted in 44 candidate protein substrates including the 15 known protein substrates of PTP1B.

**Figure 2:**
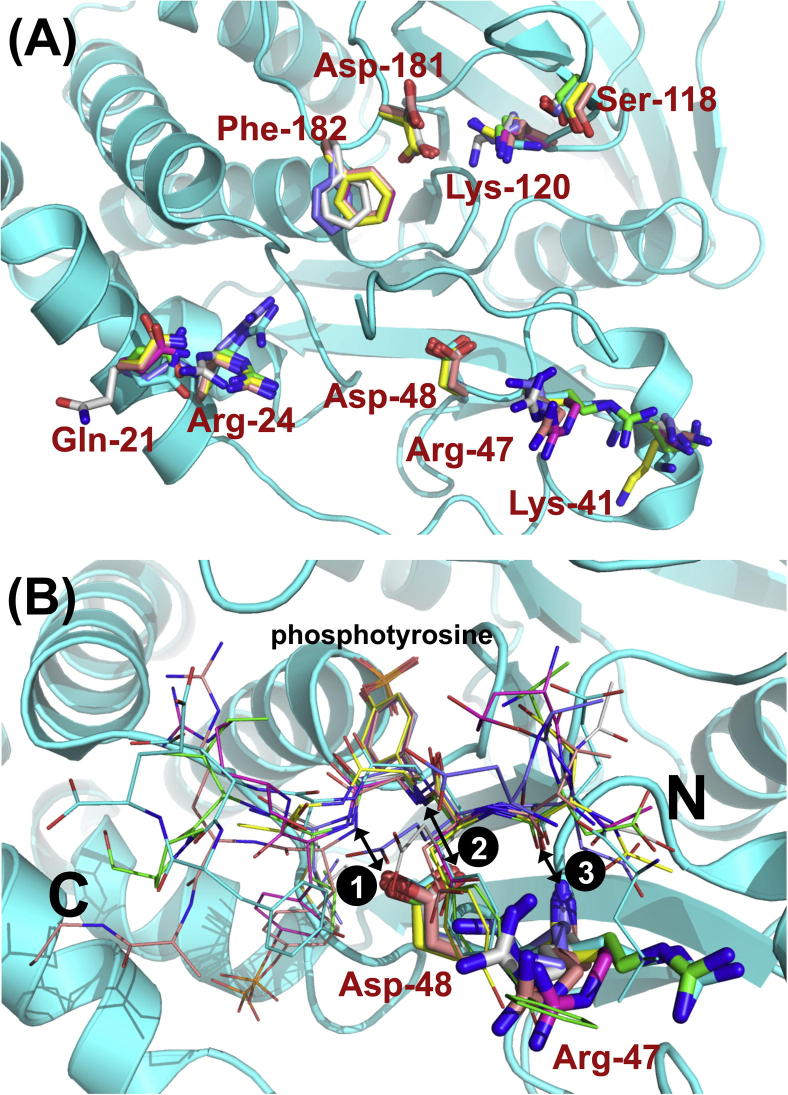
Superimposed complex structures between PTP1B and peptide substrates: PTP1B from 1EEO^19^ is shown as cartoon, the residues surrounding the active site are shown as sticks and peptide substrates are shown as lines. The carbon atoms are colored with following scheme: 1EEN (green), 1EEO (cyan), 1G1F (magenta), 1G1G (yellow), 1G1H (pink), 1PTT (gray), 1PTU (blue). (A) The residues surrounding the active site of PTP1B show a different extent of flexibility in complex with different peptide ligands; (B) The N–C terminal orientation of peptides and three common hydrogen bonds formed between peptides and PTP1B—H-bonds 1 and 2 between the Asp-48 carboxylate side chain and the main chain nitrogens of the pY and residue 1 on peptides, H-bond 3 between the main chain nitrogen of Arg-47 and the main chain carbonyl of residue-2 on peptides.

In order to refine the prediction result, of the predicted 29 novel substrates of PTP1B 35 non-redundant 11-mer peptides with single pY sites and 7 with double pY sites were extracted from original protein sequences and converted to 3D chemical structures. Then these peptide structures were docked into the PTP1B catalytic site by GOLD^17^ and the docking solutions were further refined by FlexPepDock in Rosetta.^18^ After that, the top docking solutions were manually investigated to check if they satisfy the following criteria: (i) the phosphotyrosine points into the catalytic site; (ii) the N-C terminal orientation accords with reported ones; (iii) formation of at least two of the three key hydrogen bonds^19^ (Fig. 2). After this procedure, the docking solutions of 8 peptides were found to satisfy these criteria (see Table 1, peptides 1–5, plus two peptides from JAK1 and one from Fyn listed in the Supplementary Table S1).

To test if the predicted phosphopeptides would indeed be in vitro substrates of PTP1B, we synthesized six of these peptides. The two peptides from JAK1 were not synthesized and tested because the sequences are highly similar to the ones of the known substrates JAK2^20^ and MET^21^ that were also detected by our procedure. Kinetic analysis of the corresponding JAK2 sequence KEpYpYKVK yielded similar values as obtained for the peptides tested here (*K*_m_ = 12.7 μM; *k*_cat_/*K*_m_ = 23.2 × 10^−5^ M^−1^ s^−1^),^22^ showing that these type of peptide sequences are dephosphorylated by PTP1B in vitro. With the exception of the Fyn peptide, which showed poor solubility, we then tested the enzymatic activity of PTP1B against the synthesized peptides using the in vitro EnzChek phosphate assay^23^ (Table 1). We also synthesized and tested a positive control peptide from Src as known substrate of PTP1B,^3^, ^14^ and a negative control peptide which PTP1B does not dephosphorylate.5e We found that the five peptides from the prediction were equally good substrates for PTP1B as the Src peptide, whereas as expected the negative control was not recognized. We also tested a peptide from our prediction (peptide 8 in Table 1), which was assigned low scores by all the three sequence-based methods, and found that PTP1B recognized it poorly. These results demonstrate that our protocol can successfully identify novel peptide substrates of PTP1B, and that it can differentiate between good and poor peptide substrates.

## 3 Conclusion

The here described computational approach lead to the discovery of five novel phosphopeptide substrates for PTP1B. These substrates can serve to develop substrate-based PTP1B inhibitors and other peptide-based tools.^3^ In this case the step of applying biologically meaningful filters could potentially be skipped. The here-identified new peptide substrates are derived from known phosphorylation sites on human proteins and biologically meaningful filters were applied in the procedure. Through this procedure we found 15 known protein substrates of PTP1B. Together, this suggests that the peptides can be used to search for potential physiological substrates of PTP1B. However, since we also detected JAK1, which is not a natural substrate of PTP1B, the candidates must be carefully studied in order to establish if they are natural substrates. The example of Src, which was not identified by our procedure, shows that our procedure is not exclusive; meaning that among the hits listed in Supplementary Table S1 potentially other substrate candidates can be found. Furthermore, we identified correct dephosphorylation sites for 12 of 15 protein substrates, and for the remaining 3 proteins the dephosphorylation sites are unknown, suggesting that the dephosphorylation sites on these proteins could be predicted by the phosphopeptides that we identified by our method. In addition, we expect that our protocol can be applied to other protein tyrosine phosphatases with known substrate dephosphorylation sites and protein structure data as a cheap alternative to peptide libraries and microarrays. Finally, our approach should also be applicable to other enzymatic posttranslational protein modifications (PTMs) if there is (1) available known PTM site information (sequence and position) and adequate data to construct the positive and negative data set used for the sequence-based prediction model; (2) applicable biological meaningful filters such as enzyme substrate scope or pathway involvement; and (3) available experimental structures or reliable structure models of enzymes or enzyme-substrate complexes for the molecular docking study.

## 4 Experimental section

### 4.1 Computational methods

For the method 1 with information content-based score, the sequence logo was produced from the multiple alignment of the known dephosphorylation sites of PTP1B (5 amino acids before and after the pY site) via WebLogo.^24^, ^25^ Then scores were assigned to query pY-containing peptides via adding up the height value of amino acid *i* at the *j*th position (−5 to −1 and 1 to 5) in the sequence logo.

For the PSSM score method, three amino acid frequency matrices (21 rows and 10 columns each) were generated: (i) the positive matrix value at row *i* and column *j* is the frequency of amino acid *i* (including blank where applicable) at the *j*th position (−5 to −1 and 1 to 5) in the multiple alignment of the known dephosphorylation sites of PTP1B (11-mer with 5 amino acids before and after the pY site); (ii) an analogous negative matrix which is based on the alignment of all the tyrosine sites (11-mer) from human proteins without any pY site annotation; (iii) a total matrix which is based on the alignment of all the 11-mer peptides in positive and negative matrices. Then the PSSM scores were assigned to query pY-containing peptides by adding up the overall peptide positions according to the formula in Figure 1.

Musite, which is a stand-alone application for phosphorylation site prediction, was adapted to predict dephosphorylation sites via customized prediction model training. We combined the protein sequences containing known dephosphorylation sites of PTP1B and human proteins without any pY site annotation together as input to train the prediction model. The positive and negative training sets are known dephosphorylation sites and the remaining tyrosine sites, respectively. A query protein sequence is given to this prediction model and the possible dephosphorylation sites on the protein are predicted.

For molecular docking procedure the PTP1B structure was taken from PDB entry 1EEO.^19^ 200 docking solutions were generated for each peptide by GOLD. During the docking process, the following residues in catalytic site were treated as flexible: Gln-21, Arg-24, Lys-41, Arg-47, Asp-48, Ser-118, Lys-120, Asp-181, and Phe-182. The top 10 docking solutions were sent to FlexPepDock for further refinement and 10 × 200 docking solutions were generated for each peptide.

### 4.2 Peptide synthesis

All chemicals were obtained from Sigma–Aldrich (Steinheim, Germany) and VWR (Darmstadt, Germany) and used without further purification. Fmoc-protected amino acids and Rink amide AM resin (200–400 mesh, 0.7 mmol/g) were purchased from Novabiochem (Darmstadt, Germany). The following Fmoc protected l-amino acids were used: Fmoc-Asn(Trt)-OH, Fmoc-Gln(Trt)-OH, Fmoc-Glu(*t*Bu)-OH, Fmoc-Gly-OH, Fmoc-Leu-OH, Fmoc-Lys(Boc)-OH, Fmoc-Pro-OH, Fmoc-Tyr(PO(OBzl)OH)-OH, Fmoc-Ala-OH, Fmoc-Phe-OH, Fmoc-Ile-OH, Fmoc-Arg(Pbf)-OH, Fmoc-Ser(tBu)-OH, Fmoc-Thr(tBu)-OH, Fmoc-Val-OH, Fmoc-His(Trt)-OH, Fmoc-Cys(Trt)-OH and Fmoc-Asp(*t*Bu)-OH. 2-(1H-Benzotriazole-1-yl)-1,1,3,3-tetramethyluronium hexafluorophosphate (HBTU) and Triisopropylsilane (TIS) were also purchased from Novabiochem. HPLC–MS analysis and HPLC purifications were carried out on a Shimadzu High Performance Liquid Chromatograph/Mass Spectrometer LCMS-2010EV with an UV/Vis Photodiode array detector SPD-M20A Prominence. The solvent mixtures were H_2_O and MeCN with 0.05% TFA added. RP-HPLC analytical runs were carried out with a Macherey Nagel C18 EC 250/4.0 NUCLEODUR 100-5 C18 ec column and a pump rate of 1.5 ml/min. For semi-preparative separations a Macherey Nagel C18 VP 250/10 NUCLEODUR 110-5 C18 ec column and a pump rate of 5 ml/min was used. Mass spectra were recorded on a MALDI micro MX mass spectrometer (Waters, Manchester, UK) equipped with a reflectron analyzer, used in positive ion mode with delayed extraction activated.

Solid-phase peptide synthesis (SPPS) was performed as described before3b with standard Fmoc chemistry on Rink amide resin using an automated peptide synthesizer (Syro I, Multisyntech). Acetylation, cleavage and ether-precipitation were carried out as described before3b and the peptides were purified by HPLC. Analytical data is presented in Table 2.

### 4.3 Protein expression and purification

PTP1B was expressed and purified as described previously.^3^

### 4.4 Phosphatase assay

The in vitro EnzChek phosphate assay was carried out with the commercial kit from Molecular Probes as described previously.^23^

## Figures and Tables

**Table 1:** Synthesized peptides and their activities as substrate of PTP1B

ID	Source (gene name)	pY site	Sequence	*K*_m_ [μM]	*k*_cat_/*K*_m_ (×10^−5^ M^−1^ s^−1^)
**1**	ARHGAP5	1090	DPSDNpYAEPID	28.5 ± 5.0	11.6 ± 3.4
**2**	SKAP1	271	EEEDIpYEVLPD	14.7 ± 3.2	27.9 ± 13.9
**3**	GAB2	293	DNEDVpYTFKTP	17.1 ± 4.1	9.2 ± 1.6
**4**	ACP1	133	IEDPpYpYGNDSD	20.1 ± 4.6	8.9 ± 1.1
**5**	ITGB1	783	QENPIpYKSPIN	21.7 ± 8.9	7.1 ± 3.0
**6**	Src	530	STEPQpYQPGEN	29.1 ± 5.3	9.0 ± 3.7
**7**	—	—	KKKKpYPKK	Inactive	Inactive
**8**	NOS3	657	LGSRApYPHFCA	>300	n.d.

n.d. = not determined. Peptides are acetylated at the N-terminus and contain an amide at the C-terminus.

**Table 2:** Analytical data of the synthesized peptides

Peptide	Calculated *M*_W_	Observed *M*_W_	HPLC gradient (% MeCN in H_2_O incl. 0.05% TFA)	Retention time (min)
**1**	1355	1378.0 [M+Na]^+^	10 → 50	9.8
**2**	1470	1493.0 [M+Na]^+^	10 → 50	13.6
**3**	1448	1449.0 [M+H]^+^	10 → 50	11.2
**4**	1485	1509.9 [M+Na]^+^	10 → 50	8.5
**5**	1422	1423.2 [M+H]^+^	10 → 50	10.5
**6**	1369	1392.9 [M+Na]^+^	10 → 50	3.0
**7**	1296	1296.3 [M+H]^+^	10 → 50	1.8
**8**	1341	1342.1 [M+H]^+^	10 → 50	8.8
